# Prevention of neonatal late-onset sepsis: a randomised controlled trial

**DOI:** 10.1186/s12887-017-0855-3

**Published:** 2017-04-04

**Authors:** Gary Alcock, Helen G. Liley, Lucy Cooke, Peter H. Gray

**Affiliations:** 1 Newborn Services, Mater Mothers’ Hospital, Raymond Tce, South Brisbane, QLD 4101 Australia; 2 Mater Research Institute-The University of Queensland, South Brisbane, QLD Australia; 3 Present address: The Townsville Hospital, 100 Angus Smith Drive, Douglas, 4814 Australia

**Keywords:** Preterm, Parenteral nutrition, Late-onset sepsis, Catheter associated infections

## Abstract

**Background:**

Late-onset sepsis (LOS), defined as sepsis occurring after 48 h of age causes substantial mortality and morbidity in very low birth weight infants. Risk factors for LOS include immaturity, intravascular catheters, mechanical ventilation, and prolonged parenteral nutrition (PN). Little attention has been paid to studying the effects of PN administration methods. The aim of the study was to compare a bundle of measures for PN line management incorporating a strict aseptic technique with standard line management on LOS in very low birth weight infants.

**Methods:**

Infants <1500 g birth weight who required PN were randomised to either a bundle of a strict aseptic technique for line management together with single use intravascular catheter for PN or a standard technique. The primary outcome was the incidence of LOS in the first 28 days of life. Secondary outcomes were mortality, neonatal morbidities and developmental outcome at 12 months of age.

**Results:**

There were 126 infants in the aseptic technique group and 123 in the standard technique group. Forty (31.8%) infants in the aseptic technique group and 36 (29.3%) in the standard technique group had an episode of sepsis (*p* = 0.77). This corresponds to incidences of 15.8 and 14.2 episodes of sepsis per 1000 patient days respectively. Subgroup analyses for infants <1000 g also revealed no difference in the rate of sepsis between the intervention and control groups. (*p* = 0.43). There were no significant differences in secondary outcomes and development between the groups.

**Conclusion:**

A bundle of measures including strict aseptic technique for parenteral nutrition line management did not result in a reduction in LOS when compared to a standard technique. There is no evidence to recommend this as routine practice.

**Trial registration:**

Interdisciplinary Maternal Perinatal Australasian Collaborative Trials (IMPACT) Network, TRN registration number: PT0363. Date: 06/03/2001; Australian New Zealand Clinical Trials Registry (ANZCTR), TRN registration number: ACTRN12617000455369. Date: 28/03/2017 (retrospectively registered).

**Electronic supplementary material:**

The online version of this article (doi:10.1186/s12887-017-0855-3) contains supplementary material, which is available to authorized users.

## Background

Late-onset sepsis (LOS) defined as sepsis occurring after 48 h of age causes substantial mortality and morbidity in very low birth weight infants [1, 2]. The incidence of LOS varies amongst neonatal units from 11% to 27% for infants of very low birth weight (VLBW) [2, 3]. The morbidity and mortality of episodes of hospital-acquired sepsis are high [4], with morbidities including longer duration of mechanical ventilation and hospital stay, and delays in establishing feeding. Studies also implicate neonatal infections as one of the risk factors for adverse neurological outcomes [5].

Risk factors for LOS include immaturity, intravascular catheters, mechanical ventilation, and prolonged parenteral nutrition (PN) [6]. Importantly, some studies have suggested that PN is one of the most significant sepsis risk factors in VLBW infants [7–9], although the evidence is virtually all retrospective and has not been able to account for immaturity in the analyses. Furthermore, it remains uncertain whether PN is causal in sepsis or merely associated with the presence of a venous catheter in situ.

Strategies reported to have the most significant impact in reducing the incidence of LOS have involved bundles focussing on various combinations of hand washing, infection control practices and intravenous central line management [10–12]. Few single interventions have been reported to significantly influence LOS. Aly et al. [13] however found a reduction in LOS after the introduction of a closed medication system in comparison to a historical control group.

Little attention has been paid to studying the effects of PN administration methods. In a historical cohort study, Maas et al. [14] demonstrated a significant reduction in the incidence of catheter-related bacteraemia in neonates following the introduction of a new catheter care protocol. The protocol maximised aseptic precautions and was supported by a continuing education program. There have been randomised controlled trials investigating the use of prophylactic antibiotics to reduce the risks of PN associated sepsis [15], but we could find no randomised controlled trials investigating different methods of PN line management.

Our usual practice has been to administer PN through multiuse intravascular lines with the PN bags, syringes and tubing being changed by one nurse using a clean, no touch technique. A retrospective case-control audit in our NICU indicated that the majority of episodes of LOS in VLBW infants occurred in the first 28 days of life and after adjustment for birth weight, receiving PN was the factor associated with the greatest risk [8].

The study aimed to evaluate whether a bundle of line management measures focused on reducing the risk of contamination of PN lines would reduce the incidence of LOS in VLBW babies. The hypothesis was that the study intervention; comprised of a strict sterile technique for line changes and minimising the administration of other medications or fluids through PN lines would reduce catheter contamination and by minimising breaches would reduce the incidence of LOS when compared to the standard technique routinely used in the Neonatal Intensive Care Unit (NICU). The secondary hypothesis was that the intervention would result in an improvement in developmental outcome.

## Methods

This prospective randomised controlled trial enrolled infants managed in the NICU at the Mater Mothers’ Hospital (MMH), Brisbane. Ethics approval was obtained from the MMH Research Ethics Committee and the University of Newcastle Human Research Ethics Committee. The trial was registered with the Interdisciplinary Maternal Perinatal Australasian Collaborative Trials (IMPACT) Network (Trial registration number: PT0363) and with the Australian New Zealand Clinical Trials Registry (ANZCTR) (Trial registration number: ACTRN12617000455369 [retrospective registration]). Informed written consent was obtained from a parent prior to study enrolment.

Two techniques of PN line management were employed, designated - standard and study intervention techniques. Eligible babies were randomly allocated to each technique, using a web based randomisation program (Randomization.com: http://www.randomization.com). Randomisation sequence and block size were masked in blocks of 10 stratified by birth weight (500-999 g and 1000-1499 g). Allocation was sealed in sequentially numbered opaque envelopes prepared by an administrative staff member not involved in the study.

VLBW infants with a birth weight < 1500 g were eligible if they survived at least 48 h, and were considered by the clinical team to require PN. Infants were excluded if they were not expected to survive to randomisation, had major congenital anomalies (including any infant expected to need neonatal surgery in the first 28 days) or had early onset sepsis (positive blood or CSF culture at <48 h of life). Once the decision had been made to commence PN on eligible infants and after parental consent had been obtained, babies were randomized to one of the two methods of PN line management used in the study. These are described in Table 1 and in more detail in the Additional file 1. All other care was according to NICU routines or at the discretion of the medical staff.

**Table 1:** Methods of parenteral nutrition (PN) line management employed in the study

Standard technique	Study Intervention bundle
Parenteral nutrition (PN) line changes were performed by one nurse using a no touch sterile technique but without mask, surgical scrub, or gown and gloves	Parenteral nutrition (PN) line changes by two nurses, one performing a surgical scrub and wearing a mask, gown and gloves to prevent breach of sterility.
PN lines were used as needed for other compatible infusions such as sedatives	PN lines were not used for other infusions, unless the infusion fluids were prepared and changed using the study intervention techniques
PN lines were used for boluses of medications including antibiotics	PN lines were only used for other medications in an emergency or if no other route could reasonably be used

### Primary outcome

The primary outcome was the occurrence of LOS in the first 28 days of life.

Definite sepsis was pre-defined for the purposes of the study as the presence of a culture of blood or cerebrospinal fluid positive for a pathogenic bacterium or yeast together with two of the following: physical signs of infection (any of blood pressure instability, lethargy, temperature instability, pallor, increasing apnoea and glucose intolerance); a raised haematologic sepsis score [16] (≥ 4) from the full blood count; a C reactive protein (CRP) >10 mg/L (laboratory normal value ≤10 mg/L) or new onset thrombocytopaenia. Probable sepsis was defined as a positive blood culture plus only one of: a raised hematologic sepsis score in the absence of physical signs, new onset thrombocytopaenia or a raised CRP measurement, or physical signs of sepsis and normal laboratory investigations. For the purpose of analysis, episodes of probable sepsis and definite sepsis were combined. Blood cultures were collected by venepuncture and were not obtained from intravascular catheters already in situ.

### Secondary outcomes

Mortality, chronic lung disease (oxygen requirement >36 weeks gestation), retinopathy of prematurity, duration of parenteral nutrition, duration of mechanical ventilation and respiratory support, administration of postnatal corticosteroids, duration of hospitalisation and neurodevelopmental outcome at 12 months of age.

Data other than those required for sepsis classification were collected prospectively. These included demographic data together with items needed to score the Clinical Risk Index for Babies (CRIB) [17] and Score of Neonatal Acute Physiology (SNAP) [18]. Neonatal variables collected included duration of PN, data to verify episodes of sepsis as well as mortality and late neonatal morbidities.

Following hospital discharge, the surviving infants were assessed at the Growth and Development Unit, MMH at 12 months corrected for prematurity. A psychologist who was unaware of the infant’s study group allocation and sepsis history performed the Griffiths Mental Development Scales (General Quotient [GQ], Mean 100; Standard Deviation 12) [19]. For those infants who had a GQ <50, a score of 50 was arbitrarily assigned.

## Sample size estimation

At the MMH between 1998 and 1999 the incidence of LOS was 30% [8]. A tertiary neonatal unit employing a PN management strategy similar to the study technique between 1990 and 2002 reported the incidence of LOS to range between 3.1% and 13.2% for infants <1500 g [20]. Based on an incidence of LOS of 30%, a sample size of 250 (125 per group) was calculated to demonstrate a clinically significant reduction in the incidence of LOS from 30% to 15% with Type I and II errors set at 5% and 20% respectively.

## Statistical analyses

Demographic data were compared with categorical data being analyzed using χ^2^ analyses or Fisher’s exact tests. Continuous data were analysed using Student’s t test if normally distributed and the Mann Whitney U test for non-parametrically distributed data. Subgroup analyses were performed for episodes of LOS in infants <1000 g birth weight and for episodes of LOS that commenced whilst receiving PN. Statistical calculations were performed using Stata version 8.0.

## Results

**Fig. 1:**
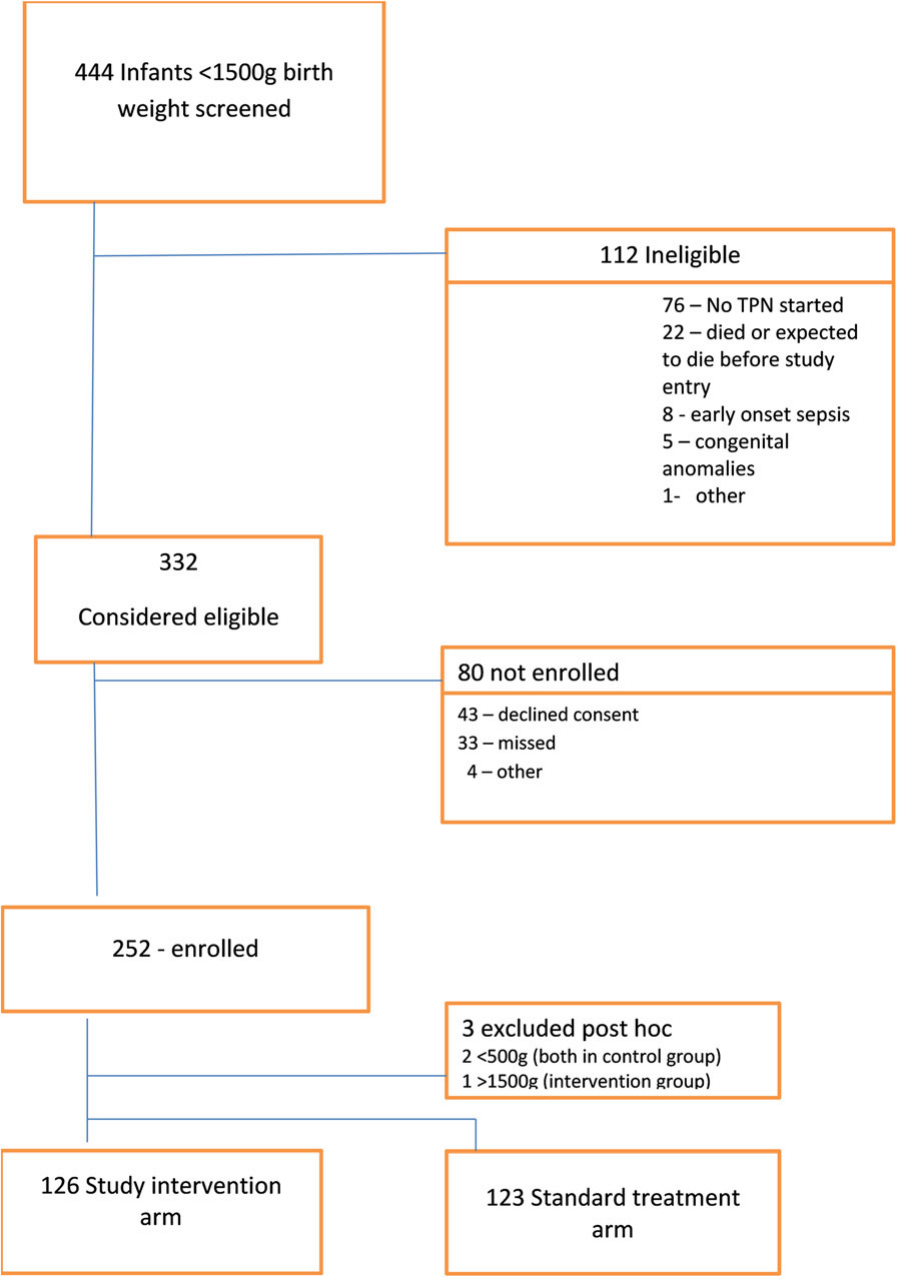
Participant flow diagram

Between May 2001 and July 2003, 444 infants of birth weight 500-1499 g were assessed for eligibility (Fig. 1). Of the 332 considered eligible, 252 infants were enrolled in the study. Of these, it was subsequently determined that three did not fulfil eligibility criteria and they were excluded post-hoc. Overall 249 randomised infants were analysed; 126 infants in the study intervention group and 123 in the standard technique group (Fig. 1). The groups were similar with respect to birth weight and gestation and other demographic characteristics (Table 2).

**Table 2:** Demographic characteristics of the study intervention bundle and standard care cohorts

	Study intervention	Standard technique
	*n* = 126	*n* = 123
Birth weight (g) – median [IQ range]	1024 [805, 1224]	1020 [795, 1210]
Infants <1000 g	61	60
Gestation (weeks) – mean (SD)	27.7 (2.4)	28.0 (2.2)
PROM	28 (22.2)	19 (15.5)
Antenatal steroids	114 (90.4)	114 (92.7)
Preterm labour	61 (48.4)	52 (42.3)
Vaginal delivery	27 (21.4)	20 (16.3)
Outborn	9 (7.1)	5 (4.1)
Surfactant	88 (69.8)	72 (58.5)
SNAP - median [IQ range]	15 [0, 32]	14 [0, 30]
CRIB score – median [IQ range]	2 [1, 6]	2 [1, 5]
Male gender	67 (53.6)	65 (53.3)
Apgar score at 5 min – median [IQ range]	8 [8,9]	8 [8,9]

Data presented as number n (%), median [interquartile range] or mean (standard deviation)

*PROM* prolonged rupture of membranes, *SNAP* Score of Neonatal Acute Physiology, *CRIB* Clinical Risk index for Babies

Four infants in the study intervention arm were withdrawn from study treatment because the protocol-driven procedures to maintain separate intravenous access for medications was considered too burdensome, but they were included in analyses on an intention to treat basis.

Seventy two infants had a positive blood culture with definite LOS, while three had probable sepsis. One infant with Serratia marcescens meningitis had a negative blood culture. Hence for analysis, 76 infants (31%) had LOS with sepsis in the first 28 days. Coagulase negative Staphylococci caused 69% of episodes of LOS during the study period, while 17% were due to gram negative bacilli. There was no difference in the distribution of organisms between the study intervention and standard care arms in the study (*p* = 0.83).

### Primary outcome

Forty infants (31.8%) in the study intervention group and 36 (29.3%) in the standard group had at least one episode of LOS in the first 28 days (Table 3). This corresponds to incidences of 15.8 and 14.2 episodes of sepsis per 1000 patient days respectively.

**Table 3:** Sepsis outcomes in study intervention bundle and standard technique groups

	Study intervention bundle	Standard technique	*P* value
	*n* = 126	*n* = 123	
Sepsis in 1st 28 days	40 (31.8)	36 (29.3)	0.77
Sepsis in 1st 28 days if birth weight < 1000 g	28 (45.9)	23 (38.3)	0.43
Sepsis in 1st 28 days whilst on parenteral nutrition	33 (26.2)	31 (25.2)	0.97

Data presented as number n (%)

In subgroup analyses for infants of birth weight < 1000 g, 45.9% randomised to the study intervention bundle and 38.3% receiving the standard technique had an episode of LOS. (Table 3) The incidence rates were 23.6 per 1000 patient days and 18.9 per 1000 patient days respectively. The majority of episodes of LOS (80%) occurred whilst infants were receiving PN and corresponded to rates in the study intervention and standard technique groups of 23.1 and 23.4 per 1000 patient days respectively.

Secondary outcomes are reported in Table 4, with no statistical differences between the groups. On follow-up at 12 months corrected for prematurity, 95 (82%) of the 116 survivors in the study intervention group attended together with 95 (83%) of the 115 in the standard technique group. The mean GQ in the study intervention group was 94.8 (SD, 13.2) compared to 94.4 (SD, 15.3) in the standard technique group (*p* = 0.85).

**Table 4:** Secondary Outcomes for the study intervention bundle and standard care cohorts

	Study intervention bundle	Standard technique	*p*
	*n* = 126	*n* = 123	
Died	10 (7.9)	8 (6.5)	0.81
Chronic lung disease	40 (31.8)	31 (25.4)	0.27
Postnatal corticosteroids	6 (4.8)	3 (2.4)	0.50
ROP > stage 2	13 (10.3)	5 (4.1)	0.085
Laser treatment for ROP	7 (5.6)	6 (4.9)	1.0
Duration of PN (days)	9.9 [5.7, 16.6]	9.0 [5.8, 14.3]	0.38
Duration of ventilation (days)	3 [0, 8.6]	2.0 [0, 7.2]	0.15
Duration of respiratory support (days)	12.9 [3.0, 39.8]	9.6 [4.0, 35.7]	0.47
Length of hospital stay (days)	73.5 [55, 92]	69 [51, 85]	0.15

Data presented as number n (%), median [interquartile range]

*ROP* retinopathy of prematurity, *PN* parenteral nutrition

## Discussion

The bundle of PN line management strategies used in the current study incorporating measures that involved a strict aseptic technique to avoid contamination during line changes together with reductions in line breaches was not associated with a reduction in the incidence of LOS when compared to standard PN line management. The prespecified subgroup analyses, while having some limitations with regard to reduced statistical power showed no benefit for the study intervention while the infants were receiving PN or for those of birth weight < 1000 g. Furthermore, there was no benefit in terms of improved developmental outcome at 12 months of age. However, with the proviso that four infants had the study intervention ceased because it was construed by clinicians as too burdensome for these patients, there were no adverse effects attributed to the intervention technique.

The results of our study were unexpected and the reasons for the lack of effectiveness of the study intervention remain unclear. It is possible that awareness among staff in the NICU that management of PN lines may affect risk for LOS might have improved diligence with respect to management of all PN lines, affecting LOS incidence in the control group.

Several studies have implicated PN as an important risk factor for LOS [7–9, 21]. The present study confirmed that most episodes of LOS in premature neonates requiring PN coincide temporally while the babies are receiving PN. It is possible that the association of PN with LOS is not causative and that the need for PN is a marker of the vulnerability for these fragile infants. This may be because infants receiving PN tend to have more invasive procedures such as placement of intravascular catheters and blood sampling.

There is strong evidence that contamination of the catheter hub is an important mechanism for entry of pathogenic bacteria into the blood stream [22], which may occur during PN line changes or during injections of medication. Thus the technique used to disinfect the catheter hub may be more important than infection control measures such as sterile gowns and gloves in preventing hub contamination. In our study, in both the study intervention and standard technique groups the catheter hub was disinfected with a 70% isopropyl alcohol swab.

It has been shown that intravenous lipid emulsions, which form an essential part of PN, affect immune function [23]. Thus PN could exert an additional risk for LOS in premature neonates through its effects on the already immature immune system of the premature infant. Hence the effect of a change in line management technique might be limited. Limitation of the use of PN and the duration of its use could be more important [24] and indeed it has been suggested that PN should be ceased and long lines removed when 120 ml/kg/day of enteral nutrition is attained [25].

Recently, it has been shown that the use of bundles of measures focussed on multiple aspects of hand hygiene, infection control measures and intravascular central line management have resulted in a reduction in the incidence of LOS in neonates [11, 12, 26]. A recent multicentred study has also shown that with a bundle of quality improvement measures there was a greater than 50% reduction in infection rates in neonatal intensive care units [25]. Unfortunately, comparisons with the current study are not possible as Bowen et al. [25] reported infection rates for their total population, whereas our infection rates relate only to infants in the study who were receiving PN. The evidence from previous studies is limited in that infection rates are compared with a historical cohort, but the results are consistent and have led to recommendations for the prevention of infection in the NICU [27]. There are likely to be many sources of infection in preterm infants. Any beneficial effects of one intervention may be obscured by the other reservoirs of infection. Our bundle of measures was designed to reduce the contamination of PN line catheter hubs during line changes. However, despite best efforts, lines could become contaminated via the skin breach or by haematogenous seeding.

There was little evidence that the study intervention was harmful although the study intervention technique was abandoned in four infants (3.2%) because of difficulty in inserting peripheral intravenous lines (PIVs) for intravenous medication or infusions. The use of a PIV for medication injections in addition to the intravenous line for PN infusion however was successfully accomplished in the majority of babies.

It has been shown that preterm infants with infection during the neonatal period are more likely to have neurodevelopmental impairment including cognitive development than for uninfected infants [28, 29]. In the present study, given that there was no difference in LOS between the study arms, it was not surprising that there was no difference in developmental outcomes between the two groups of infants. The developmental assessment however occurred at 1 year of age with no opportunity for assessment at an older age. If later developmental assessments had been performed, it is possible that differences in cognitive development may have become evident.

Limitations to the study include the fact that the study was undertaken more than a decade ago during which time there have been a number of changes in practices in many neonatal units that may reduce the rate of LOS. These include the use of probiotics and the more rapid rate of enteral feed increases resulting in shorter periods of PN. Nevertheless, given that the intervention arm focussed on PN line changes, it is felt that the study provides important information relevant to current management. A difficultly in the diagnosis of neonatal infection is that it is usual practice to withdraw blood from a peripheral vein for culture and not from central catheters. Thus it is possible that rates of sepsis could be underestimated or indeed overestimated as contamination with skin organisms especially coagulase negative staphylococci is a possibility. The diagnosis of infection in our study was rigorous and required additional evidence of sepsis including physical signs and abnormal laboratory results. Thus it is considered that the rate of infection documented in the present study is likely to be accurate.

The study, however had many strengths including blinding of outcome assessment, intention to treat analysis, relatively large sample size, and the low rate of attrition. Criteria for the primary outcome measure (LOS episodes) and other outcomes were predefined. Although the study was randomised, there were some minor weaknesses. Three infants were withdrawn from the study after randomisation. This was due to mistaken recruitment of two infants <500 g birth weight and one >1500 g, who were not eligible for inclusion. The inadvertent inclusion of these three infants was not a systematic error and post randomisation exclusion was considered appropriate.

## Conclusion

The bundle of PN line management employing a two-nurse sterile technique and measures to reduce breaches of lines used to infuse the PN did not reduce the incidence of LOS in infants of <1500 g birth weight. There was no evidence from this study to recommend this as a routine practice. Further research is required to assess measures that are potentially effective to reduce what is considered to be a preventable complication of prematurity.

## Additional file

Additional file 1: Parenteral nutrition line management techniques. Description of data: Details of the parenteral nutrition line change management – study intervention bundle and standard technique. (DOCX 20 kb)

## Abbreviations

CRIB: Clinical Risk Index for Babies
CRP: C-reactive protein
GQ: General Quotient
LOS: Late-onset sepsis
MMH: Mater Mothers’ Hospital
NICU: Neonatal Intensive Care Unit
PIV: Peripheral intravenous line
PN: Parenteral nutrition
ROP: Retinopathy of prematurity
SNAP: Score of Neonatal Acute Physiology
VLBW: Very low birth weight
