# Supplementary material for: Prevention of neonatal late-onset sepsis: a randomised controlled trial
Source: BMC Pediatr. 2017 Apr 4;17:98. doi: 10.1186/s12887-017-0855-3 (PMC5381090; doi:10.1186/s12887-017-0855-3)
Supplement: Additional file 1: — Parenteral nutrition line management techniques. Description of data: Details of the parenteral nutrition line change management – study intervention bundle and standard technique. (DOCX 20 kb) [file 12887_2017_855_MOESM1_ESM.docx]

**Additional file 1 Parenteral nutrition line management techniques**

**Parenteral Nutrition Line Change Management – Study Intervention Bundle**

All infusion changes to parenteral nutrition (PN) lines performed by two nurses, one wearing mask and sterile gown and gloves and using a strict aseptic technique.

# **Frequency of Line Change**

Amino Acid/Dextrose changed every 48-72 hours.

Intralipid/Vitamin solution dispensed by the pharmacy in syringes changed every 24 hours.

Medication (continuous infusion) changed every 24 hours.

Clear fluid (e.g. 0.9% sodium chloride) changed every 72 hours.

# **Procedure**

1. All Amino Acid/Dextrose and Intralipid/Vitamin line changes carried out using the ‘Study Intervention’ technique with lines not used for other infusions or bolus injections, unless fluids prepared and changed using the ‘Study Intervention’ technique.
2. At line changes, the hubs were disinfected with a 70% isopropyl alcohol swab.
3. Second intravenous line preferred to administer any other intravenous medications.
4. PN lines used for other medications only in an emergency, or if no other route could reasonably be used. If the PN lines were used for other medications, then these were prepared using the ‘Study Intervention’ technique, unless an emergency necessitated otherwise.
5. 3-way taps only fitted to PN infusion lines if other infusions (e.g. sedation) were administered concurrently.
6. Filters (in-line) – Pall Neo 96^®^ (Pall Life Sciences, Port Washington, NY) used for PN lines.
7. A SmartSite^®^ (Smith Medical, ST Paul, MN) directly connected to all venous catheters and venous cannula extension tubing and left in situ at line change.
8. Double lumen UVC – one lumen used for infused PN fluid and the other lumen used for blood products and medications. Each lumen considered to be a separate line, so when the PN was infused though one lumen it was accessed only using the ‘Study Intervention’ technique, with the other lumen accessed as per the ‘Current Standard of Care’ line change policy.

## Parenteral Nutrition Line Change Management – Standard Technique

All infusion line changes performed by using a careful aseptic technique.

# **Frequency**

Amino Acid/Dextrose Solution changed every 48-72 hours

Intralipid/Vitamins (syringe and line) changed every 24 hours

Medication (continuous infusion) changed every 24 hours

Clear fluids (e.g.10% Dextrose with or without additives) changed every 72 hours, or if prescription changes.

# **Procedure**

- At line changes, the hubs were disinfected with a 70% isopropyl alcohol swab.

1. Pall Neo 96^®^ filter used for parenteral nutrition to filter particulates and microbes.
2. Intralipid/Vitamins not filtered.
3. SmartSite^®^ connected directly to all venous catheters and/or venous extension tubing, this left in situ at line change.
4. 3-way tap – all infusion lines had a 3-way tap at the distal end, with a SmartSite^®^ on the side port which was then connected to the catheter/cannula SmartSite^®^ and was changed with the line change.
5. Double lumen UVC – one lumen used for the infused fluids, and the other for blood products or medications.
6. Where no arterial line was available, the second UVC lumen was infused with heparinised 0.9% sodium chloride to enable venous blood gases to be taken.
7. Where a peripheral intravenous catheter in situ, this was used for intravenous medications (using standard aseptic technique)
